# Valganciclovir for cytomegalovirus viraemia in advanced HIV disease: a phase 2b randomized placebo-controlled trial of valganciclovir for cytomegalovirus viraemia in adults and adolescents with advanced HIV disease

**DOI:** 10.1098/rstb.2024.0402

**Published:** 2025-11-06

**Authors:** Jayne Ellis, Laura Joan Nsangi, Mohammed Rassool, Joseph Musaazi, David S. Lawrence, Caleb Skipper, David R. Boulware, David B. Meya, Sean Wasserman, Joseph N. Jarvis, Tom Boyles

**Affiliations:** ^1^ Department of Clinical Research, London School of Hygiene and Tropical Medicine, London WC1E 7HT, UK; ^2^ Infectious Diseases Institute, Makerere University, Kampala, Uganda; ^3^ Clinical HIV Research Unit, a division of the Wits Health Consortium, Helen Joseph Hospital, Johannesburg 2193, South Africa; ^4^ Botswana Harvard Health Partnership, Gaborone, Botswana; ^5^ School of Pathology, Faculty of Health Sciences, University of the Witwatersrand, Johannesburg, South Africa; ^6^ Department of Medicine, University of Minnesota, Minneapolis, MN 55455, USA; ^7^ School of Medicine, College of Health Sciences, Makerere University, Kampala, Uganda; ^8^ Institute for Infection and Immunity, City St George's University of London, London EC1V 0HB, UK; ^9^ Wellcome Discovery Research Platforms in Infection, Centre for Infectious Diseases Research in Africa, Institute of Infectious Disease and Molecular Medicine, University of Cape Town, Cape Town, South Africa

**Keywords:** valganciclovir, CMV viraemia, cytomegalovirus, RCT, HIV

## Abstract

Mortality among adults hospitalized with advanced HIV disease (AHD; CD4 <200 cells μl^−1^ or a WHO stage 3 or 4 disease) is >20%; there is an urgent need to evaluate strategies to reduce mortality within this extremely high-risk group. Approximately 50% of adults with CD4 counts <100 cells μl^−1^ have detectable cytomegalovirus (CMV) viraemia, and high-level CMV viraemia is associated with greater than double the hazard of death in this population. Despite this, there are no current treatment guidelines to inform treatment of CMV viraemia without end-organ disease in the context of AHD. The NIRVANA study is a double-blinded, placebo-controlled, multi-centre phase 2b trial to evaluate efficacy and safety of valganciclovir for patients with AHD and CMV viraemia. We will enrol 150 hospitalized adults and adolescents (aged ≥15 years) with CD4 <100 cells μl^−1^, and CMV viraemia >500 IU ml^−1^ from two hospitals in Uganda and South Africa. Participants will be randomized 1 : 1 to either valganciclovir 900 mg orally or matched placebo for 28 days. Safety monitoring and CMV viral load testing will occur weekly until week 4, and thereafter at week 8 and at study termination at week 12. The primary endpoint is a composite of adverse events of special interest, re-hospitalization or death within 8 weeks. Secondary endpoints include median reduction in CMV viral load at day 28 and week 12, proportion with CMV below the limit of detection at weeks 2, 4, 8 and 12, mortality, grade ≥3 adverse events and serious adverse events, duration of hospitalization, tolerability, HIV treatment response, proportion with ganciclovir resistance and valganciclovir pharmacokinetics. Data from the NIRVANA phase 2 trial including valganciclovir pharmacokinetic data will be used to inform design of a multi-site phase 3 randomized controlled trial powered for survival to investigate whether valganciclovir reduces mortality among hospitalized adults with AHD-associated CMV viraemia.This article is part of the discussion meeting issue ‘The indirect effects of cytomegalovirus infection: mechanisms and consequences’.

## 1. Background

Despite widespread availability of antiretroviral therapy (ART), human immunodeficiency virus (HIV) remains in the top five causes of mortality in sub-Saharan Africa, predominately due to advanced HIV disease (AHD) [1,2]. It is estimated that more than 4 million people have AHD—defined as having a CD4 <200 cells μl^−1^ or a WHO stage 3 or 4 disease—and each year more than 600 000 of these are expected to die [3,4]. The risk of death is highest among hospitalized persons with AHD, in whom in-patient mortality consistently exceeds 20% [5]. Strategies are urgently needed to reduce mortality within this extremely high-risk group.

Cytomegalovirus (CMV) is a ubiquitous herpes virus. Following early childhood exposure, near-universal seroconversion to CMV is seen by 5 years of age in most low-resource settings [6]. Reactivation of latent CMV infection, with active CMV replication, is common in AHD, and up to 50% of adults with CD4 counts of <100 cells μl^−1^ have detectable CMV viraemia [7–9]. CMV reactivation is highly immunogenic and is associated with CMV-induced T-cell differentiation and upregulation of type 1 interferons (IFNs). Type 1 IFNs have important antiviral activity but downregulate type 2 IFNs, such as IFN-γ produced by type 1 T-helper cells responding against intracellular pathogens, including *Cryptococcus* and *Mycobacterium tuberculosis*. In theory, this downregulation may increase susceptibility to, and disease severity of, these opportunistic infections. These *indirect effects* of CMV infection occur irrespective of the presence of CMV end-organ disease and partly underpin the rationale for prophylaxis, or pre-emptive treatment of CMV viraemia [10].

Observational studies have repeatedly shown that CMV viraemia is associated with worse short- and medium-term outcomes in the context of AHD, including an increased risk of AIDS-defining events, tuberculosis and death [7–9,11,12]. A prospective cohort study of 374 people living with HIV (PWH) with serial CMV measurements, found that CMV viraemia was significantly associated with increased progression to a new AIDS-defining illness (relative rate 2.22; 95% CI, 1.27−3.88), and death (relative rate 4.14; 95% CI, 1.97−8.70)), and these associations were independent of both CD4 count and HIV viral load [11]. Among 811 adults with AHD and cryptococcal meningitis recruited into the AMBITION-cm trial, CMV viraemia occurred in half of all participants, and high-level CMV plasma viraemia (>1000 IU ml^−1^) was associated with double the odds of mortality at 2 weeks (adjusted odds ratio (aOR), 2.31; 95% CI, 1.12−4.75) and 10 weeks (aOR 2.44; 95% CI, 1.33−4.45) after adjustment for potential confounders including ART and CD4 count. Data from AMBITION-cm participants also demonstrated a dose−response relationship between increasing CMV viraemia and death [13]. Observational studies, however, have been unable to demonstrate a causal relationship between CMV viraemia and poor outcomes, and it remains plausible that CMV viraemia in the context of AHD is a non-pathogenic marker of immunosuppression—a so-called ‘bystander infection’ [10]. An interventional trial of CMV treatment to determine whether treatment of CMV viraemia improves outcomes in AHD is therefore required.

There are no current treatment guidelines to inform treatment of CMV viraemia among PWH. International HIV guidelines currently highlight the importance of early ART initiation rather than anti-CMV therapy for the management of CMV viraemia [4,14,15]. However, in the context of AHD among hospitalized adults, where one in five will die during admission, irrespective of ART status, an alternative strategy is needed. The optimal treatment for CMV viraemia in this group is to be determined; specifically, whether valganciclovir, the most widely available CMV anti-viral, is a safe and effective treatment needs to be evaluated. We hypothesize that short-course anti-CMV therapy with valganciclovir to treat CMV viraemia prior to established ART and immune reconstitution may improve outcomes.

Valganciclovir is a readily available anti-CMV therapy, which is frequently used for the treatment of HIV-associated CMV end-organ disease [16], and for both anti-CMV prophylaxis, and pre-emptive anti-CMV therapy for patients undergoing solid organ or stem cell transplantation [17]. It has not previously been evaluated as a treatment for AHD-associated CMV viraemia in the absence of CMV end-organ disease. The potential benefits of treating CMV viraemia within this group are clear [10]; however, the dose-dependent bone marrow suppression associated with valganciclovir may make its use prohibitive and its safety needs to be evaluated within a phase 2 trial; pharmacokinetic data to inform dosing are lacking in this patient population. Safety and virological efficacy data from the NIRVANA phase 2 trial, including valganciclovir pharmacokinetic data, will be used to inform the design of a multi-site phase 3 randomized controlled trial (RCT) powered to determine whether valganciclovir reduces mortality among hospitalized adults with AHD-associated CMV viraemia.

### (a) Study design

The NIRVANA study is a double-blinded, placebo-controlled, multi-centre trial to evaluate efficacy and safety of valganciclovir for patients with advanced HIV disease and CMV viraemia. Participants will be randomized 1 : 1 to either valganciclovir 900 mg orally or matched placebo for 28 days.

## 2. Study objectives

The primary objective is to determine if valganciclovir is safe and efficacious in reducing CMV viraemia among hospitalized adults with advanced HIV disease and CMV viraemia.

Secondary objectives are to determine the effect of valganciclovir on mortality, to study its pharmacokinetics and to explore the immunological response of patients with CMV viraemia before and after treatment with valganciclovir.

### (a) Study population and setting

The NIRVANA study will recruit 150 hospitalized adults and adolescents (aged ≥15 years) with CD4 <100 cells μl^−1^ and CMV viraemia >500 IU ml^−1^ from two hospitals in Uganda and South Africa: Mulago National Referral Hospital, Kampala, Uganda, and Helen Joseph Hospital, Johannesburg, South Africa. The first 20 participants at the Uganda site will all receive open-label valganciclovir and contribute to an intensive pharmacokinetic sub-study to describe valganciclovir pharmacokinetics in the context of AHD. The subsequent 130 participants across both sites will contribute to the RCT.

## 3. Hypothesis

Our hypothesis is that valganciclovir, provided at study entry at 900 mg once daily for 4 weeks, will reduce CMV viral load compared with placebo, and be a safe therapeutic option for the treatment of AHD-associated CMV viraemia.

## 4. Study endpoints

The NIRVANA trial primary endpoint is a composite of adverse events of special interest plus re-hospitalization or death within 8 weeks. The NIRVANA adverse events of special interest are absolute neutrophil count <0.4 × 10^9^ l^−1^; haemoglobin <7 g dl^−1^; platelets <50 × 10^9^ l^−1^; creatinine increases to >1.5× participant’s baseline; alanine transaminase (ALT) >5 × upper limit of normal (ULN).

The secondary endpoints are:

(1) Adverse events of special interest plus re-hospitalization or death up to week 12
(2) Hospital length of stay post randomization
(3) Serious adverse events (SAEs) up to week 12
(4) Mortality: Survival time and all-cause mortality at 4 and 12 weeks
(5) Efficacy:
  (I) Median reduction in CMV viral load (log_10_ IU ml^−1^) from baseline to day 28 and week 12
  (II) Proportion with CMV below the limit of detection at weeks 2, 4, 8 and 12
(6) Tolerability: Proportion of participants who prematurely discontinue trial medication
(7) Ganciclovir resistance: Genotypic resistance to ganciclovir at week 4 by sequencing the UL97 kinase UL54 DNA polymerase genes in all who remain viraemic >1000 IU ml^−1^ at day 28
(8) HIV treatment response: Proportion with HIV viral suppression (<50 copies ml^−1^) at 12 weeks stratified by ART status at baseline
(9) Valganciclovir pharmacokinetics
(10) Incident CMV end-organ disease.

### (a) Screening and consent

#### (i) Inclusion criteria

Consecutive hospitalized adults and adolescents (≥15 years) will be screened during admission for NIRVANA study eligibility. Participants must be living with HIV, have a CD4 count <100 cells μl^−1^, and CMV viraemia >500 IU ml^−1^. All participants must provide written informed consent for screening, and trial inclusion.

#### (ii) Exclusion criteria

Patients with confirmed or high level of clinical suspicion for CMV end-organ disease including CMV retinitis will be excluded, as will patients with any of the following blood abnormalities: ALT >3 × ULN; estimated creatinine clearance <40 ml min^−1^; absolute neutrophil count <1.0 × 10^9^ l^−1^; haemoglobin <8 g dl^−1^; and platelets <100 × 10^9^ l^−1^. Patients receiving high-dose acyclovir or valacyclovir will be excluded, as will patients receiving highly myelosuppressive medications, e.g. amphotericin deoxycholate B and linezolid. Patients who are unable to swallow the investigational tablets will be excluded from the study. Pregnant and lactating women will also be excluded. Patients with a previous serious reaction to valganciclovir, patients expected to die within the next 48 h, and patients who are expected to be unable to complete 12 weeks of follow-up will also be excluded.

#### (iii) Consent

Written informed consent to enter the trial will be obtained from participants or, in the case of those lacking capacity to consent, surrogate consent from next of kin with legal responsibility can be provided. Adolescents (aged ≥15–17 years) must provide written assent to inclusion, and additionally parent/guardian consent must be provided. Participants who have capacity to consent but are unable to sign for themselves will be asked to have a witness present (friend, family, or another member of staff independent of the study team) to witness the discussion and thumbprint consent.

The aims, implications, potential benefits and risks associated with the study will be explained in full to all potential participants and/or the next of kin. It will be made clear to potential participants that refusal to participate in the study will not jeopardize their clinical care, and it will be made clear that consent is entirely voluntary and can be withdrawn at any time. Participants enrolled via surrogate consent will be re-consented as soon as their mental status improves and they regain the capacity to consent, with care taken to ensure they understand that they are free to withdraw from the study and if they do so this will not jeopardize their future care. Original signed consent forms will be kept by the investigator; participants will be given a copy of the signed/thumb-printed consent form and a participant information sheet.

### (b) Randomization and treatment allocation

Following screening and enrolment, participants will be randomly assigned in a 1 : 1 ratio to receive either valganciclovir or placebo using a computer-generated block randomization method with a block size of 4 ensuring an equal distribution of participants to each group within each block. Randomization will be stratified by site and CMV viral load >500–1000 and >1000 IU ml^−1^.

The randomization sequences will be created using REDCap. The system will automatically assign participants to the next available slot in the sequence upon their enrolment in the study, ensuring allocation concealment. The allocation sequence will not be disclosed to the participants, investigators or study staff responsible for participant recruitment and data collection. Both the participants and the study staff will be blinded to the treatment assignments.

### (c) Study interventions

**Figure 1.**
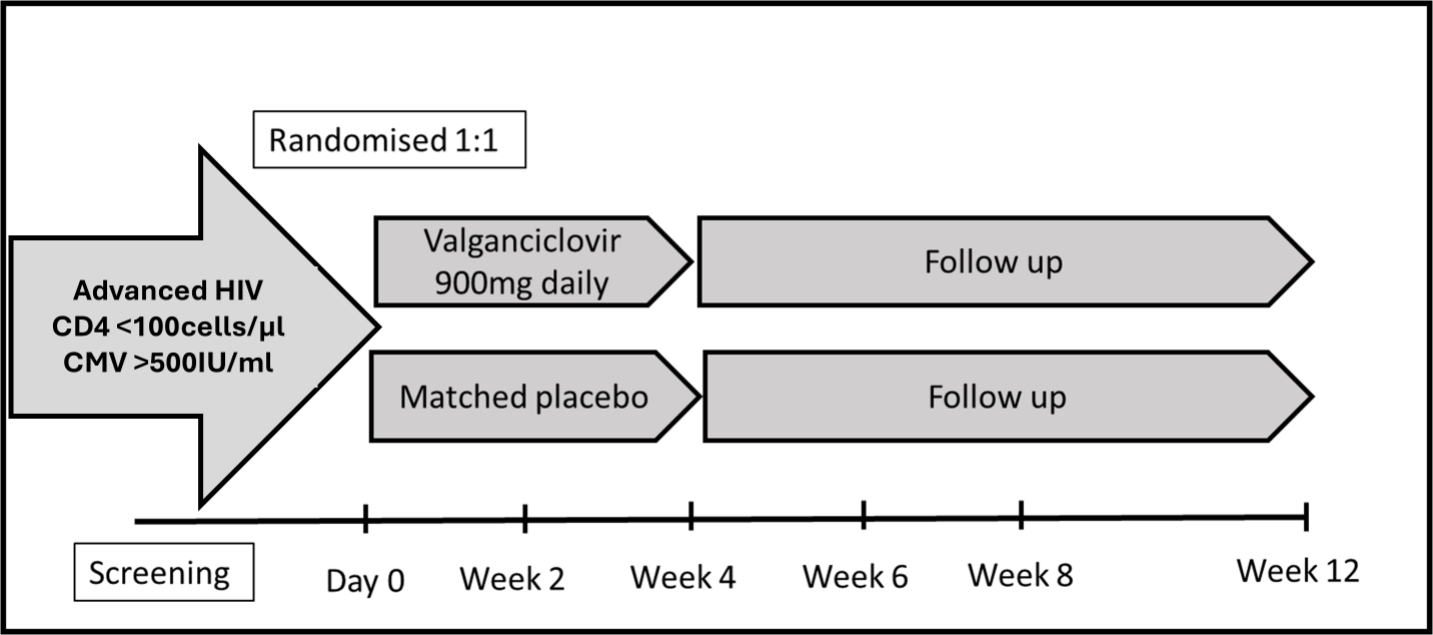
NIRVANA trial schema.

Participants will be randomized to either valganciclovir 900 mg once daily orally, starting at the time of randomization and lasting for 28 days, or matching placebo, at the same dosing schedule (figure 1). Participants with estimated glomerular filtration rate (eGFR) 40−60 ml min^−1^ will be dosed at 450 mg daily. Adolescents aged 15 years and older will follow the adult dosing protocol used for individuals aged 18 years and above. The dose of valganciclovir—900 mg per day—is selected to balance expected efficacy with toxicity risk (particularly cytopenias); pill burden, tolerability, cost and feasibility of delivering twice daily regimens were also considered. This dosing regimen is aligned with recommendations for prophylaxis in solid organ transplantation, where a dose of 900 mg day^−1^ is given [17]. A duration of 28 days was selected to cover the time taken to initiate ART for most participants—such that valganciclovir is provided as a therapeutic bridge to management of CMV viraemia until effective ART-associated immune reconstitution has been achieved. Our chosen approach aims to suppress CMV viraemia during the highest-risk window, while minimizing the risks and costs associated with longer treatment courses. Furthermore, there is limited, but supportive, evidence for our dosing strategy in AHD. Valganciclovir provided at 900 mg daily for 8 weeks did not result in more serious adverse events nor increased risk of cytopaenias in a small RCT involving 14 participants living with HIV on ART.

The valganciclovir and placebo tablets will be identical in appearance, packaging and administration schedule, thus maintaining the double-blind nature of the study.

Study medication will be administered by a ward nurse with the assistance of study staff while patients are in hospital and self-administered upon hospital discharge. Study drugs will be dispensed for one week at a time.

The trial will be conducted on a background of standard of care (SOC) according to relevant guidelines (national and/or local) which is expected to include: diagnosis and treatment of opportunistic infections; antiretroviral therapy; tuberculosis (TB) preventive therapy; cotrimoxazole prophylaxis; and cryptococcal antigen screening plus antifungal pre-emptive therapy if positive. All SOC interventions will be provided through routine care.

#### (i) Participant follow-up

Participants will have a full history and examination at time of enrolment to include: demographics, presenting complaints and signs, ART history and concomitant medications review. Following commencement of assigned study drugs, participants will undergo regular clinical assessment including physical examination to monitor for any adverse events (table 1). Study participants will be reviewed daily by the NIRVANA study team while admitted.

**Table 1.** NIRVANA schedule of events table. POC, point of care; CBP, child-bearing potential; CMV, cytomegalovirus; ALT, alanine transaminase; PK, pharmacokinetics.

	SCR	day 0	day 4	day 7	day 14	day 21	day 28	week 8	week 12	unscheduled visit
visit window in days	−3		±1	±1	±1	±2	±2	±3	±3	
written informed consent	X									
eligibility assessment	X									
randomization		X								
prescription for treatment		X								
participant reimbursement	X	X	X	X	X	X	X	X	X	X
*clinical evaluation*										
symptoms & physical examination	X	X		X	X	X	X	X	X	X
adverse events assessment			X	X	X	X	X	X	X	X
medication review		X		X	X	X	X	X	X	X
*urine*										
POC pregnancy test for women of CBP	X									

*blood*										
HIV testing	±									
HIV viral load									X	
CD4 count	X								X	
CMV viral load	X				X		X	X	X	
ALT and bilirubin	X	X		X	X	X	X	X	X	X
complete blood count	X	X		X	X	X	X	X	X	X
creatinine	X	X		X	X	X	X	X	X	X
plasma storage for ganciclovir resistance	±						±			

*pharmacokinetics*										
PK sparse sampling			X	X	X		X			

*immunology*										
storage for serum cytokines		X					X			

Participants may be discharged at the discretion of the attending study physician when medically fit. Time of hospital discharge following consent and randomization will therefore vary and will depend on the clinical condition of the patient. At time of hospital discharge, participants will be provided with the mobile number of the NIRVANA study team. Participants will be encouraged to attend for review with the NIRVANA study doctor if they experience symptomatic deterioration.

Following hospital discharge, protocolized participant follow-up including clinical review, safety monitoring bloods, pharmacokinetic (PK) sampling and CMV viral load evaluation will be conducted in the NIRVANA study outpatient clinic. In-person follow-up appointments will be weekly from week 1 through to week 4; following valganciclovir completion in-person follow-up appointments will occur at weeks 8 and 12.

#### (ii) Pharmacokinetic sampling

Sparse PK sampling to measure plasma valganciclovir concentration will occur for all randomized participants on study days 4, 7, 14 and 28. Sampling will occur at 2−3 h post-dose to coincide with peak vGCV concentrations (*C*_max_). Height, weight and body mass index (BMI) will be captured at each PK visit.

#### (iii) Safety monitoring blood tests

Full blood count including differential, creatinine, ALT and total bilirubin will be tested at study days 7, 14, 21 and 28 and weeks 8 and 12. Any participant with adverse events of special interest while on treatment will be referred to the study clinician for clinical evaluation within 24 h of the results being known. The study clinician will determine whether the medication should be stopped or continued.

CD4 and HIV viral load will be tested at baseline, and re-checked at the week 12 study visit.

#### (iv) Cytomegalovirus viral load monitoring

CMV viral load testing by quantitative polymerase chain reaction (qPCR) will be conducted at baseline, and at weeks 2, 4, 8 and 12 study visits.

CMV qPCR will be performed on plasma specimens in accordance with a local CMV viral load standard operating procedure (SOP). DNA quantification will be reported in IU ml^−1^ to standardize across sites. Assays will adhere to rigorous quality assurance (QA)/quality control (QC) protocols to ensure accuracy and reliability. Pre-analytical controls verify sample integrity and reagent quality. Analytical procedures include instrument calibration, use of internal controls and validation of standard curves to ensure precise quantification. Post-analytical quality checks ensure data consistency, and results are reviewed against internal and external controls. Ongoing monitoring includes proficiency testing, operator training and regular audits. Any deviations trigger corrective actions, ensuring continuous compliance with regulatory standards and accurate patient results. This approach guarantees reliable viral load measurements for effective clinical management.

#### (v) Valganciclovir genotypic resistance testing

Among participants with detectable CMV viraemia >1000 IU ml^−1^ at day 28, we will perform valganciclovir genotypic resistance testing. In participants with evidence of valganciclovir genotypic resistance at day 28, we will further test the baseline plasma samples. The results of the valganciclovir genotypic resistance testing will enable us to investigate if there is evidence of increased genotypic resistance to valganciclovir among participants who receive treatment with valganciclovir versus those who receive placebo for the treatment for CMV viraemia. All valganciclovir genotypic resistance testing will be conducted using stored samples.

#### (vi) Anti-retroviral therapy management

Most patients will not be taking effective ART at the time of admission to hospital. Most will be eligible as per World Health Organization (WHO) guidelines [4] to (re-)initiate effective ART within 2 weeks of admission. Individuals with cryptococcal and TB meningitis will not start ART until they have completed 4−8 weeks of meningitis treatment owing to risk of paradoxical immune reconstitution inflammatory syndrome (IRIS). ART initiation, re-initiation or switch will be done in conjunction with the participant’s ART clinic in accordance with national guidelines.

### (d) Statistical methods

Analysis plans for primary and secondary outcome measures will be addressed in the statistical analysis plan (SAP), to be finalized prior to database lock. In brief, the primary endpoint will be analysed using a hierarchical ordinal scale, using the intention-to-treat population. The analysis comparing the virological efficacy of valganciclovir versus control will include all randomized participants using geometric mean ratio (GMR) of log_10_CMV with 95% confidence intervals. Secondary analyses will use the same approach. For safety analyses, counts and percentages of adverse events will be presented by randomized group and absolute risk differences will be presented with confidence intervals.

A total of 122 patients (61 per arm) are required to provide the study with 80% power to detect a statistically significant difference in the primary outcome between the treatment and control groups, using a two-sided significance level of 5% (α = 0.05). This calculation is based on detecting an increase in the proportion of patients achieving the primary outcome from 20% in the control group (placebo) to 43% in the experimental group (valganciclovir). Following consultation with key stakeholders, these proportions reflect a clinically meaningful effect size. The calculation accounts for standard assumptions of binomial proportions in two independent groups. Total sample size for the RCT is therefore 130 (65 participants per group) to account for dropouts.

### (e) Ancillary studies

#### (i) Intensive pharmacokinetics sub-study

Data on the pharmacokinetics of valganciclovir in adults with AHD are lacking. Data generated by the NIRVANA intensive PK sub-study will be used to inform valganciclovir treatment recommendations for the treatment of AHD-associated CMV viraemia. Knowledge of valganciclovir PK is mainly from paediatric patients and those with solid organ or stem cell transplantation. There is wide variability in exposures, influenced by bioavailability (absorption and prodrug conversion) and clearance (renal function), which may be influenced by patient factors in AHD.

At the Uganda site only, a lead-in intensive PK sub-study of 20 participants receiving 900 mg orally once daily for 28 days will be conducted prior to commencement of the randomized NIRVANA trial. In Uganda, the NIRVANA RCT will only commence once the lead-in intensive PK sub-study has been completed.

The purpose of the intensive PK sub-study is to allow intensive PK sampling only among participants receiving valganciclovir (i.e. not those receiving placebo). The reason for this separate cohort is that the phase II trial is double-blinded, and it would be inappropriate to conduct intensive PK sampling on individuals not receiving the study medication.

##### Intensive pharmacokinetics sub-study screening and enrolment procedures

Inclusion and exclusion criteria for the NIRVANA intensive PK sub-study are the same as for the NIRVANA RCT. Screening procedures for the intensive PK sub-study will be aligned with screening for the parent NIRVANA RCT. Participants must be living with HIV, have a CD4 count <100 cells μl^−1^ and CMV viraemia >500 IU ml^−1^, and have none of the listed exclusion criteria. The first 20 eligible participants in Uganda will be approached for consent for inclusion into the intensive PK sub-study. Potential participants will be given a specific PK sub-study patient information sheet, and the purpose, conduct, benefits and risks of being screened will be explained. Written informed consent will be obtained from all participants. There is no surrogate consent option for the intensive pharmacokinetic sub-study, and adolescents (aged <18 years) will not be eligible for the intensive PK sub-study.

##### Intensive pharmacokinetics sub-study procedures and follow-up

All participants included in the intensive PK sub-study will receive valganciclovir 900 mg orally once daily for 28 days. No participants will receive placebo. Participants will have directly observed valganciclovir administration, and thereafter undergo intensive PK sampling to measure valganciclovir concentration in the plasma at the following time points: pre-dose, and at 1, 2, 3, 4, 6 and 8 h post valganciclovir dose. All PK sampling will take place at the day 4 study visit, after attainment of steady-state plasma concentrations. Participants in the intensive PK sub-study will have the same follow-up through week 12, and the same safety monitoring as randomized NIRVANA participants, but will not form part of the randomized trial group for analysis.

##### Intensive pharmacokinetics sub-study analysis plan

Ganciclovir concentrations will be determined by liquid chromatography and tandem mass spectrometry. All samples will be analysed at the Infectious Diseases Institute PK laboratory in Kampala, Uganda. Data from the intensive PK sub-study will be analysed by non-compartmental methods to describe secondary PK parameters. In addition to non-compartmental analysis, we will use nonlinear mixed effects modelling to describe total ganciclovir plasma concentrations from intensive and sparse sampling, using the final model to predict individual exposures to explore relationship with treatment effect markers (CMV viral load and cytopenia).

### (ii) Immunology sub-study

To explore the immunological mechanisms that might drive the deleterious effects of CMV viraemia, we will perform two baseline immune assays on all individuals randomized into the trial. Blood samples will be collected at baseline and on day 28 for planned immunology sub-studies.

First, we will perform quantification of serum cytokines using a Luminex multiplex magnetic bead assay. We will use R&D Biosystems 21-plex premixed T-cell panel assay (R&D Systems, Minneapolis, MN, USA)—which focuses on T-helper cell type 1 versus type 2 responses (Th1 versus Th2). The samples will be run in duplicate to ensure a coefficient of variance of <10%. Our primary analysis approach will be to perform principal component analysis. We will test for associations between the component cytokines and baseline CMV viral load, prediction of rate of CMV viral suppression, development of opportunistic infection and survival. We will also determine the changes in the cytokine profile from baseline to day 28, comparing results for those receiving valganciclovir versus placebo.

Second, we will perform a commercial IFN-γ release assay (IGRA) enzyme-linked immunosorbent assay (ELISA) (QuantiFERON-CMV; Qiagen, Germantown, MD, USA). We hypothesize that quantitative IFN-γ release after CMV antigen stimulation might predict risk in persons with CMV viraemia and AHD. To perform the assay, whole blood is collected in three separate tubes (mitogen, CMV antigen, nil) and incubated overnight. Plasma is then spun off and cryopreserved. Subsequently, an ELISA is used to quantify the amount of IFN-γ produced on the batched samples. We will determine if a positive CMV IGRA is associated with improved survival in persons with AHD and CMV viraemia using *χ*^2^-testing. We can also assess if the quantitative IFN-γ concentration is associated with time to viral suppression and survival via linear and/or logistic regressions.

### (f) Ethical approval

The trial will commence when investigators have obtained approval from the following research ethics committees: Mulago Hospital Institutional Review Board, University of the Witwatersrand, and London School of Hygiene & Tropical Medicine. Approvals will also be obtained from the Uganda National Council of Science and Technology (UNCST), Ugandan National Drug Authority (NDA) and South African Health Products Regulatory Authority (SAHPRA). Any further amendments will be submitted and approved by each ethics committee, and communicated with all study investigators prior to implementation.

### (g) Quality control and assurance

Trial oversight will be provided by the Trial Management Group (TMG), Trial Steering Committee (TSC) and Independent Data Safety Monitoring Committee (DSMC). The trial is sponsored by the Wits Health Consortium, and hosted by the Infectious Diseases Institute, Uganda and the Clinical HIV Research Unit, Helen Joseph Hospital, Johannesburg, South Africa. The sites will be monitored at regular intervals with visits by the trial monitor in order to monitor the conduct of the trial and ensure that the principles of International Conference of Harmonization (ICH) Good Clinical Practice (GCP) are being adhered to. Visits will ensure that all training has been completed, that drug supply and equipment are in place and that all staff are up to date on the protocol and procedures.

### (h) Data collection and management

Data will be collected on electronic case report forms (eCRFs) using an electronic data capture system; paper CRFs/worksheets may be used to capture information for periods when electronic data capture is unavailable. It is the investigator’s responsibility to ensure the accuracy, completeness, legibility and timeliness of the data reported on the participant’s eCRF. All data will be kept secure, and confidentiality of all study participants will be carefully protected. Data will be validated, managed and stored in a de‐identified database on a secure server at the Clinical HIV Research Unit in Johannesburg, South Africa.

### (i) Sample use and storage

Consent forms also include consent for storage of samples (blood and urine) in accordance with Uganda National Council for Science & Technology guidelines and London School of Hygiene and Tropical Medicine (LSHTM) Human Tissue Act Policy. Participant specimens will be stored for current and future research studies related to advanced HIV disease, opportunistic infections including CMV and the immune system in accordance with local standards and LSHTM Human Tissue Act Policy.

### (j) Confidentiality

All participant-related information will be kept strictly confidential. Participants will be identified only by means of a coded number specific to each participant. All computerized databases will identify participants by numerical codes only and will be password-protected. Consent forms will be stored under the supervision of each local primary investigator (PI) in a secured office and accessible to trial staff only.

### (k) Dissemination of results

We will share results through presentations at scientific conferences and in peer-reviewed open-access journals.

## 5. Discussion

The NIRVANA trial will provide critical safety and virological efficacy data to inform valganciclovir use for the treatment of CMV viraemia in the context of AHD. Data from the NIRVANA trial, including valganciclovir PK data, will be used to inform design of a subsequent multi-site phase 3 RCT to evaluate whether pre-emptive treatment of CMV viraemia with valganciclovir reduces mortality amongst hospitalized adults with AHD. Valganciclovir is the most readily available and affordable anti-CMV drug in regions of the world where the majority of persons with AHD are cared for. The potential impact of demonstrating that valganciclovir is safe and efficacious for the treatment of CMV viraemia in this very high-risk population would be a substantial reduction in AHD-related mortality globally.

## Data Availability

This article has no additional data.
